# Determinants of growth measurements in rural Cambodian infants: a cross-sectional study

**DOI:** 10.1093/inthealth/ihaa018

**Published:** 2020-05-07

**Authors:** Asuka Miyazaki, Mitsuaki Matsui, Rathavy Tung, Bunsreng Taing, Laura V White, Azusa Iwamoto, Sharon E Cox

**Affiliations:** School of Tropical Medicine and Global Health, Sakamoto 1-12-4, Nagasaki University, Nagasaki 852-8523, Japan; School of Tropical Medicine and Global Health, Sakamoto 1-12-4, Nagasaki University, Nagasaki 852-8523, Japan; National Maternal and Child Health Centre, Ministry of Health, France street, Phnom Penh, Cambodia; Kampong Cham Provincial Health Department, Ministry of Health, Preah Kosamak Nearyroth, Kampong Cham, Cambodia; School of Tropical Medicine and Global Health, Sakamoto 1-12-4, Nagasaki University, Nagasaki 852-8523, Japan; Bureau of International Health Cooperation, National Centre for Global Health and Medicine, Toyama 1-12-1, Shinjuku-ku, Tokyo 162-8655, Japan; School of Tropical Medicine and Global Health, Sakamoto 1-12-4, Nagasaki University, Nagasaki 852-8523, Japan; Institute of Tropical Medicine, Nagasaki University, Sakamoto 1-12-4, Nagasaki 852-8523, Japan; London School of Hygiene and Tropical Medicine, Keppel Street, London WC1E 7HT, UK

**Keywords:** Cambodia, hygiene, infants, undernutrition, malnutrition, growth

## Abstract

**Background:**

Inappropriate feeding and hygiene practices and poor environment are associated with malnutrition. We aimed to investigate the contributions of feeding, hygiene practices and recent illness to the nutritional status of rural Cambodian infants and any sex-specific differences.

**Methods:**

In a cross-sectional study, nested within an ongoing birth cohort, trained fieldworkers conducted anthropometry and collected information from the main caregiver during home visits. Multivariable linear regression was used to investigate associations with nutritional status as length-for-age z-scores (LAZ) and weight-for-length z-scores (WLZ).

**Results:**

A total of 156 children, 87 (55.8%) male, ages 0–11 months were enrolled. The prevalence of acute malnutrition (WLZ <−2) in males and females was 2.3% (2/87) and 5.8% (4/69), respectively, and 23.0% (20/87) of males and 14.5% (10/69) of females were stunted (LAZ <−2). WLZ but not LAZ decreased with age. WLZ was independently negatively associated with increasing age (β-coefficient −0.14 [95% confidence interval {CI} −0.20 to −0.08], p<0.001), and regular use of feeding bottles (β-coefficient −0.46 [95% CI −0.83 to −0.10], p=0.014), and positively with handwashing with soap (β-coefficient 0.40 [95% CI 0.05 to 0.75), p=0.027).

**Conclusions:**

The prevalence of acute malnutrition was low, but stunting was prevalent without evidence of a sex difference. Non-linear growth faltering was associated with increasing age and hygiene/feeding practices.

## Introduction

The ‘First 1000 Days’ movement stresses the long-term importance of nutrition during the first 1000 d of a child’s life, from conception to 2 y of age .^1^^,^^2^ Undernutrition is an important underlying cause of child mortality and negatively affects children’s physical and cognitive development, and is associated with future productivity.^2^^,^^3^ Chronic undernutrition results in linear growth faltering or ‘stunting’, while acute undernutrition results in reduced weight gain or weight loss, assessed by comparison with a reference healthy population of the same age deemed to have ‘optimal’ growth.^4^ Globally, among children <5 y of age, approximately 149 million are estimated to be moderately or severely stunted and 50 million have moderate or severe acute undernutrition (frequently termed moderate/severe malnutrition [MAM/SAM]).^5^ In Cambodia, increasing economic growth has contributed to improving child nutritional status, yet the prevalence of stunting, acute undernutrition and low weight for age in children <5 y of age remains high at 32%, 10% and 24%, respectively.^6^

**Figure 1:**
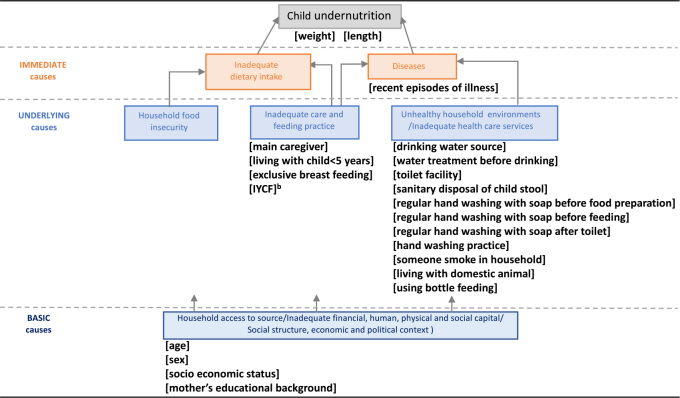
Conceptual framework of factors associated with undernutrition assessed in this study^a^ Variables included in this study are shown in brackets. ^a^Figure modified from UNICEF conceptual framework (Improving child nutrition: the achievable imperative for global progress. New York: UNICEF; 2013). ^b^IYCF: infant and young child feeding indicators. (World Health Organization. Indicators for assessing infant and young child feeding practices. Part 1: definitions. Geneva: World Health Organization; 2008).

The causes of undernutrition are multifactorial. The United Nations Children’s Fund (UNICEF) has developed a framework to describe determinants contributing to maternal and child undernutrition, grouped into immediate causes of inadequate nutrition intake and infection; underlying causes of household food insecurity, inadequate child care and feeding practices; inadequate access to health services and unhealthy environment; and basic causes relating to the socio-economic and political context (Figure 1).^7^^,^^8^ The introduction of nutrient-poor complementary foods during weaning can result in inadequate nutrient intake and increased risk of infections through unsafe food and water, unclean utensils and unhygienic food preparation and environment. Frequent infections result in increased nutrient and energy requirements and reduced nutrient absorption and gastrointestinal infections,^9^^,^^10^ all of which can contribute to a downward spiral leading to undernutrition.^11^ In Cambodia, only 30% of the population in rural areas have access to improved sanitation and 69% of the population have access to improved drinking water.^12^

In a preparatory survey of infant nutritional status conducted to inform the design of the ongoing birth cohort, known as ‘Nutrition for Health of Aka-chan (baby in Japanese) and Mamas’ (NHAM cohort), in Kampong Cham province in Cambodia, the prevalence of moderate acute malnutrition (weight-for-length z-score [WLZ]<−2) was significantly higher in male compared with female infants (15.9% [22/138] vs 8.3% [9/109], p=0.018) with growth faltering appearing from 6 months of age (unpublished data, Iwamoto et al. 2017). There is conflicting evidence for differences in undernutrition by sex, which may vary by context and the specific age group.^13–17^ Sex-specific differences in children’s living environments and care practices, as well as biological factors,^18–20^ may contribute to sex-specific differences in the development of undernutrition.

A better understanding of the underlying factors related to suboptimal growth and undernutrition, especially poor sanitation and hygiene in children’s living environment and if there are important differences by sex, is required to design better nutrition-sensitive interventions to improve child growth. Therefore we aimed to determine the prevalence of undernutrition in rural Cambodian children <1 y of age and if this differed by sex, and to explore potential behavioural and environmental causes of child undernutrition, as shown in Figure 1**.**

## Materials and methods

### Study setting

This was a substudy within the NHAM cohort study conducted in 12 rural villages in the communes of Khbop Ta Nguon, Preah Andoung and Peam Koh Sna, in Kampong Cham province, located in southeast Cambodia (Supplementary Figure A). The NHAM cohort study registered all infants born between April 2016 and March 2019, and is following them until 2 y of age to investigate the causes of stunting.

### Study design

This was a cross-sectional study in which all children <1 y old and participating in the NHAM cohort study were eligible for recruitment.

### Data collection

Data collection occurred between 16 March 2017 and 11 April 2017. Four days of training was provided to all the fieldworkers prior to the survey. Trained fieldworkers, with a certificate in nursing, midwifery or medicine, visited households and collected data using a paper-based questionnaire. The questionnaire collected birthweight; demographic characteristics of the parents or main caregiver of the child; household assets; water, sanitation and hygiene facilities and practices; infant feeding practices^21^^,^^22^ and recent history of illness episodes (last 14 d), including diarrhoea, fever and cough. A standardized socio-economic status (SES) score was generated from household facility and assets information using principal components analysis and analysed as quintiles. The minimum dietary diversity score, minimum meal frequency and consequent minimum acceptable diet for children ≥6 months of age were calculated as per the formulae for infant and young child feeding practice (IYCF) indicators^21^^,^^22^ as used previously in the Cambodia Demographic and Health Survey 2014.^6^ Sanitation and hygiene facilities and practices of households, such as drinking water source, water treatment, toilet facility of the household and disposal of the child’s stools, were assessed as improved/unimproved, sanitary/unsanitary or adequate/inadequate by core questions on drinking water and sanitation for household surveys.^6^^,^^23^ The questionnaire was initially created in English and translated into the local language (Khmer) by an experienced bilingual, medically qualified research assistant. It was then pilot tested with all fieldworkers in English and Khmer in discussions with the investigator, and revisions were finalized before implementation.

### Anthropometry measurements

The weight and length of all children were determined by fieldworkers using a weighing scale (Seca 877, Seca, Hamburg, Germany) and a length board (Seca 417, Seca). The weight and length of each child were measured to the nearest 0.1 kg and 1 mm, respectively.

### Sample size

Sample size was determined by the parent NHAM cohort and no sample size calculation was done for this substudy. A total of 184 children born since 1 April 2016, age <1 y and who lived in the target villages were enrolled in the NHAM cohort by the end of February 2017. All were eligible for this substudy.

### Data analysis

All collected data were entered into EpiData software (EpiData Association, Odense, Denmark). WLZ and length-for-age z-score (LAZ) values were calculated using the World Health Organization (WHO) Anthro software (version 3.2.2). Moderate acute malnutrition (MAM) is defined as WLZ ≥−3 and <−2, severe acute malnutrition (SAM) as WLZ <−3 and stunting as LAZ <−2. Data analysis was conducted using Stata14.1 (StataCorp, College Station, TX, USA). The prevalence of MAM/SAM and stunting by age and sex were calculated and compared using χ^2^ tests. Differences in mean WLZ and LAZ by sex were tested by Student’s t-test. Linear regression was used to investigate associations between WLZ and LAZ as continuous measures with explanatory independent variables. Multivariable linear regression modelling was carried out using a forward stepwise approach. Sex and age were included a priori. Independent variables were selected to be taken forwards for possible inclusion in the multivariable model if the association in the univariable analysis had a likelihood ratio test (LRT) p-value <0.2. Variables were considered in blocks of related exposures, where blocks comprised demographic/SES proxies, hygiene-related factors, recent reported illness/symptoms and smoking. Models were compared at each step using LRTs ,and independent variables were retained in the model if the LRT p-value was <0.05.

## Results

A total of 184 children born since 1 April 2016, <1 y of age and enrolled in the NHAM cohort were eligible for this substudy, which represented 81.7% of all recorded singleton births in the study areas during this period. Of the 184 NHAM participants, 28 were not found at home and therefore 156 children were enrolled in this substudy. Eighty-seven participants were male (55.8%). The basic characteristics of the children by sex are shown in Table 1. There were no differences by sex for any of the variables except the educational background of the mother. Only 39 (25.0%) households used an improved water source for drinking and 96 (61.5%) households used improved toilet facilities. While 126 (80.8%) of the main caregivers answered that they washed their hands with soap after going to the toilet, only 60 (38.5%) answered that they washed their hands with soap before food preparation.

**Table 1:** Participants and household characteristics (N=156)

Variables		All	Sex		p-Value
			Male	Female	
Total, n (%)		156	87 (55.8)	69 (44.2)	
Age group (months), n (%)	0–5	82 (52.6)	42 (48.3)	40 (58.0)	NS^a^
	6–8	41 (26.3)	25 (28.7)	16 (23.2)	
	9–11	33 (21.2)	20 (23.0)	13 (18.9)	
Birthweight (g), mean (SD)		3086 (430)	3124 (421)	3037 (440)	NS^b^
Low birthweight (<2500 g), n (%)		8 (5.1)	4 (4.6)	4 (5.8)	
Nutritional status	WLZ, mean (SD)	−0.03 (1.20)	0.01 (1.18)	−0.08 (1.23)	NS^b^
	WLZ<−2, n (%)	6 (3.8)	2 (2.3)	4 (5.8)	NS^a^
	LAZ, mean (SD)	−1.14 (0.96)	−1.24 (0.95)	−1.03 (0.98)	NS^b^
	LAZ<−2, n (%)	30 (19.2)	20 (23.0)	10 (14.5)	NS^a^
Mother’s level of education^c^, n (%)	No formal education	9 (5.9)	7 (8.1)	2 (3.0)	0.03^a^
	Some primary	42 (27.6)	20 (23.3)	22 (33.3)	
	Completed primary	25 (16.5)	13 (15.1)	12 (18.2)	
	Some secondary	60 (39.5)	41 (47.7)	19 (28.8)	
	Completed secondary and more	16 (10.5)	5 (5.8)	11 (16.7)	
Socio-economic status (quintile), n (%)	Lowest	31 (19.9)	13 (14.9)	18 (26.1)	NS^a^
	Second lowest	32 (20.5)	21 (24.1)	11 (15.9)	
	Middle	31 (19.9)	15 (17.2)	16 (23.2)	
	Fourth	30 (19.2)	20 (23.0)	10 (14.5)	
	Highest	32 (20.5)	18 (20.7)	14 (20.3)	
Main caregiver is mother, n (%)		138 (88.5)	76 (87.4)	62 (89.9)	NS^a^
Living with other children <5 y of age in the household, n (%)		53 (34.0)	24 (27.6)	29 (42.0)	NS^a^
Smoking inside the household, n (%)		71 (45.5)	39 (44.8)	32 (46.4)	NS^a^
Improved drinking water source^c^, n (%)		39 (25.0)	24 (28.2)	15 (21.7)	NS^a^
Regular water treatment before drinking, n (%)		91 (58.3)	52 (59.8)	39 (56.5)	NS^a^
Improved toilet facility in the household, n (%)		96 (61.5)	55 (63.2)	41 (59.4)	NS^a^
Sanitary disposal of childrens’ stool, n (%)		68 (43.6)	40 (46.0)	28 (40.6)	NS^a^
Regular handwashing with soap before food preparation, n (%)		60 (38.5)	37 (42.5)	23 (33.3)	NS^a^
Regular handwashing with soap before feeding, n (%)		47 (30.1)	26 (29.9)	21 (30.4)	NS^a^
Regular handwashing with soap after going to the toilet^c^, n (%)		126 (80.8)	71 (82.6)	55 (79.7)	NS^a^
Using feeding bottle with nipple, n (%)		78 (50.0)	46 (52.9)	32 (46.4)	NS^a^
Childrens’ living environment (exposure to domestic animal), n (%)	Not living with domestic animals	77 (49.4)	44 (50.6)	33 (47.8)	NS^a^
	Living with domestic animals, separated from place where child spends time	59 (37.8)	33 (37.9)	26 (37.7)	
	Living with domestic animals, not separated from place where child spends time	20 (12.8)	10 (11.5)	10 (14.5)	
One or more reported episodes of illness in previous 2 weeks^c^, n (%)	Diarrhoea	17 (10.9)	10 (12.5)	7 (10.1)	NS^a^
	Fever	34 (21.8)	13 (16.3)	21 (30.4)	NS^a^
	Cough	51 (32.7)	23 (28.8)	28 (40.6)	NS^a^

SD: standard deviation; NS: not significant.

^a^χ^2^ test.

^b^Student’s t-test.

^c^Missing data: mother’s level of education (n=4), drinking water source of the household (n=2), handwashing with soap after toilet (n=1), one or more reported episodes of illness in previous 2 weeks (diarrhoea, fever, cough) (n=7).

Fifty percent of children were reported to be fed by using a feeding bottle. Among 82 children <6 months of age, the prevalence of exclusive breastfeeding was 66.7% (54/81). In the non-exclusively breastfed children <6 months of age, 11 had received water, 1 had formula milk and 6 had other liquids or foods in the previous 24 h in addition to breast milk, while 9 were fed only formula. In all the children, 82.6% (123/149) had received breast milk in the previous 24 h. Among children ≥6 months of age, the proportion who had received a diet meeting the minimum acceptable dietary diversity score (minimum of four food groups in the previous 24 h) and minimum meal frequency was 24% (16/68). Almost all children ≥6 months of age who were not currently being breastfed had received formula milk in the previous 24 h (25/26).

**Figure 2:**
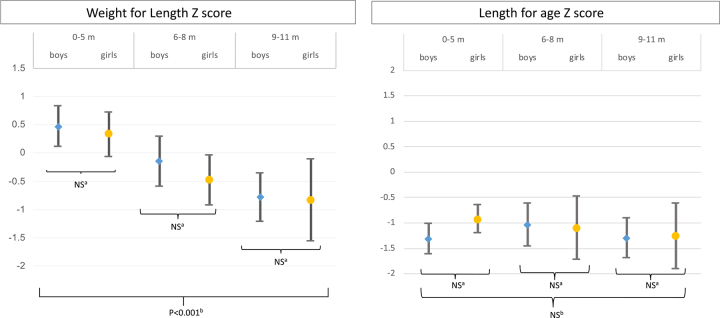
WLZ and LAZ (mean±95% CI) by age group and sex ^a^Student’s t-test by sex in age group. ^b^Analysis of variance test by age group.

The number of male and female children with MAM (WLZ<−2) was two (2.3%) and four (5.8%), respectively. There was no child with SAM (WLZ<−3). Twenty (23.0%) male and 10 (14.5%) female children were stunted (LAZ<−2), with no evidence of a difference by sex (Table 1). However, WLZ significantly decreased with age, while no association with age was observed for LAZ (Figure 2).

Table 2 describes associations between the exposure variables with child nutritional status assessed using univariable linear regression. Increasing age in months (p<0.001), reported regular use of a feeding bottle (p<0.001), one or more reported episodes of cough in the previous 14 d (p=0.004), one or more reported episodes of fever in the previous 14 d (p=0.019), lowest SES (p=0.03) and living with another child <5 y of age in the same household (p=0.05) were associated with lower WLZ. There were no associations with LAZ except for birthweight, in which normal (≥2500g) compared with low birthweight was associated with increased LAZ (p<0.001).

**Table 2:** Univariable linear regression risk factor analysis for nutritional status (N = 156)

Variables	Coefficient (95% CI)		
		WLZ	LAZ
Age (months)		−0.16^c^ (−0.22 to −0.10)	−0.01 (−0.06 to 0.04)
Sex (female vs male)		−0.08 (−0.47 to 0.30)	0.21 (−0.10 to 0.51)
Birthweight ≥2500 g		−0.24 (−1.11 to 0.62)	1.66^c^ (1.02 to 2.30)
Mother's level of education^d^	No formal education	Ref.	Ref.
	Some primary	−0.06 (−0.95 to 0.62)	−0.08 (−0.76 to 0.61)
	Completed primary	0.03 (−0.91 to 0.97)	−0.09 (−0.82 to 0.63)
	Some secondary	0.11 (−0.76 to 0.98)	−0.26 (−0.93 to 0.40)
	Completed secondary and more	0.15 (−0.86 to 1.17)	0.43 (−0.35 to 1.21)
Main caregiver is mother		0.49 (−0.10 to 1.08)	−0.25 (−0.73 to 0.23)
Living with other children <5 y old in the household		−0.40 (−0.79 to 0.00)	0.12 (−0.20 to 0.44)
Smoking inside the household		−0.27 (−0.65 to 0.11)	0.28 (−0.02 to 0.58)
Socio-economic status (lowest quintile group)		−0.52^a^ (−0.99 to −0.05)	−0.03 (−0.41 to 0.36)
Improved drinking water source^d^		0.32 (−0.12 to 0.76)	−0.19 (−0.54 to 0.17)
Regular water treatment before drinking		−0.05 (−0.44 to 0.34)	0.04 (−0.27 to 0.35)
Improved toilet facility		0.24 (−0.15 to 0.63)	0.16 (−0.15 to 0.47)
Sanitary disposal of childrens’ stool		−0.30 (−0.68 to 0.58)	0.10 (−0.20 to 0.41)
Regular handwashing with soap before food preparation		0.28 (−0.10 to 0.67)	−0.14 (−0.45 to 0.18)
Regular handwashing with soap before feeding		−0.23 (−0.64 to 0.19)	0.11 (−0.22 to 0.45)
Regular handwashing with soap after going to the toilet^d^		0.07 (−0.42 to 0.56)	0.26 (−0.13 to 0.65)
Using feeding bottle with nipple		−0.77^c^ (−1.13 to −0.41)	0.13 (−0.17 to 0.44)
Children's living environment (exposure to domestic animal)	Not living with domestic animals	Ref.	Ref.
	Living with domestic animals separated from place where child spends time	0.02 (−0.40 to 0.43)	−0.17 (−0.5 to 0.16)
	Living with domestic animals, not separated from place where child spends time	−0.02 (−0.62 to 0.58)	0.09 (−0.38 to 0.57)
One or more reported episodes of illness in previous 2 weeks^d^	Diarrhoea	−0.42 (−1.03 to 0.20)	0.10 (−0.39 to 0.59)
	Fever	−0.49^a^ (−0.89 to −0.08)	0.11 (−0.22 to 0.44)
	Cough	−0.59^b^ (−1.00 to −0.19)	0.04 (−0.30 to 0.36)

^a^p-Value <0.05.

^b^p-Value <0.01.

^c^p-Value <0.001.

^d^Missing data: mother’s educational background (n=4), improved drinking water source (n=2), regular handwashing with soap after toilet (n=1), one or more reported episodes of illness in previous 2 weeks (diarrhoea, fever, cough) (n=7).

In a subset analysis in children <6 months of age, exclusive breastfeeding was associated with higher WLZ (n=81, β-coefficient=0.78, p=0.004), which remained significant after adjusting for age (β-coefficient=0.68, p=0.020), but no association was observed for LAZ. In children ≥6 months of age with available data (n=68), there was no evidence that meeting the minimum acceptable diet was associated with either WLZ or LAZ.

In the final multivariable model for WLZ with age and sex included a priori (Table 3), increasing age (p<0.001) and regular use of a feeding bottle (p= 0.014) were associated with decreased WLZ and handwashing with soap before food preparation with an increased WLZ (p=0.027). For LAZ, with age and sex included a priori, normal birthweight remained significantly associated with increased LAZ (p<0.001), and regular handwashing after going to the toilet was positively associated (p=0.041) (Table 3).

**Table 3:** Final multivariable model of factors associated with WLZ (n=156) and LAZ (n=155)

Variables^a^		Coefficient (95% CI)
WLZ	Age (months)	−0.14^c^ (−0.20 to −0.08)
	Sex (female)	−0.14 (−0.49 to 0.20)
	Using feeding bottle with nipple for the child	-0.46^b^ (−0.83 to −0.10)
	Regular handwashing with soap before food preparation	0.40^b^ (0.05 to 0.75)
LAZ	Age (months)	−0.005 (−0.05 to 0.04)
	Sex (female)	0.25 (−0.03 to 0.53)
	Normal birthweight	1.89^c^ (1.21 to 2.57)
	Regular handwashing with soap after toilet	0.38^b^ (0.02 to 0.74)

^a^Age and sex were included a priori and all variables shown were retained in the final model as described in the statistical methods.

^b^p-Value <0.05.

^c^p-Value <0.001.

## Discussion

This study was conducted to investigate the prevalence and risk factors of child undernutrition, particularly related to infant feeding practices, the hygienic situation of households, the child’s living environment and episodes of child illness, and if these differed by sex in rural villages in Cambodia. There was a low prevalence of MAM (3.8%; 0% SAM) but a higher prevalence of stunting (19.2%). Contrary to earlier observations from the preparatory survey, there was no evidence of an increased risk of undernutrition in males compared with females in this population of children <1 y of age. WLZ was independently and negatively associated with reported use of a feeding bottle and positively associated with the main caregivers’ regular handwashing practice with soap before food preparation. Normal birthweight and main caregivers’ regular handwashing practice with soap after going to the toilet showed evidence of an association with increased LAZ.

An increased risk of stunting and underweight in male infants 0–23 months of age has been observed previously in the Cambodian population^17^ and in other populations <5 y of age in sub-Saharan Africa^13^ and South Asia.^15^ However, such a sex difference was not observed in a recent multicountry study in 20 high-burden countries, including Cambodia, assessing risk factors for acute malnutrition in children <6 months of age using national Demographic and Health Survey (DHS) data.^16^ In an analysis of children <2 y old using DHS data from Cambodia and Kenya, the prevalence of stunting was significantly more common in Kenyan boys; and, although the prevalence of stunting was also higher in Cambodian boys, the difference did not reach statistical significance.^14^ The reduced prevalence of undernutrition and the lack of difference in nutritional status by sex observed in this study compared with those observed in the previous preparatory survey is unlikely to have resulted from the season of data collection, as both were conducted in the dry season—March–April in the current study and February–March in the preparatory survey.

Our results suggest that the hygiene practices of caregivers are an important factor for child nutritional status. Feeding bottles, if not cleaned properly or if liquids are left in the bottle for a long time before consumption, can be a source of infections.^24^ As most children in this study were also receiving breast milk and the use of formula was not associated with nutritional status, we suggest that bottle hygiene practices rather than differences in nutritional intake are the most likely explanation for this observation. Our study results showed caregivers’ regular handwashing with soap before food preparation and after going to the toilet was associated with improved nutritional status. A similar result has been observed in another study in India, but particularly so for stunting, in which diarrhoeal episodes from faecal–oral contamination were also associated with poor child growth.^25^ Diarrhoeal episodes are known to be directly associated with poor child growth,^26^ while asymptomatic intestinal inflammation and environmental enteropathy have also been associated.^10^^,^^27^ In our study, the recent incidence of fever and cough, but not diarrhoea, was associated with decreased WLZ, but was no longer significantly associated when adjusted for bottle feeding and handwashing. It is possible that use of feeding bottles and lack of regular handwashing are proxies for a generally less hygienic child environment, in turn associated with more cough and fever and with mostly asymptomatic effects on environmental enteric dysfunction contributing to lower WLZs.^27^

## Study limitations

This study has several limitations. First, it is not possible to determine causality or possible seasonality due to the cross-sectional design. Second, household or caregiver’s hygiene and sanitation practices, child feeding practices and child illness episodes were not directly observed or diagnosed but were self-reported by the primary caregivers. This could result in information bias since interviewees might be more likely to give the perceived desirable responses. Finally, it was not possible to estimate the likely adequacy of nutritional intakes in this study, but instead we focused on factors that might correlate with increased exposure to infections as a cause of acute or chronic undernutrition.

## Conclusions

There was no evidence of an increased risk of malnutrition in males compared with females. The prevalence of wasting was not significantly higher than the expected prevalence in a normal distribution of 2.25%; however, 20% of children were stunted. There was evidence to support the importance of hygiene practices by caregivers in improving non-linear growth in this population. Somewhat surprisingly, no effects on linear growth were observed. Further investigation of the effects of hygiene and environmental factors on the gut microflora, function, environmental enteropathy and growth is recommended.

## Supplementary Material

ihaa018_Supplimental_Figure
