# Supplementary material for: Determinants of growth measurements in rural Cambodian infants: a cross-sectional study
Source: Int Health. 2020 May 7;13(1):49–56. doi: 10.1093/inthealth/ihaa018 (PMC7807240; doi:10.1093/inthealth/ihaa018)
Supplement: ihaa018_Supplimental_Figure [file ihaa018_supplimental_figure.docx]

**
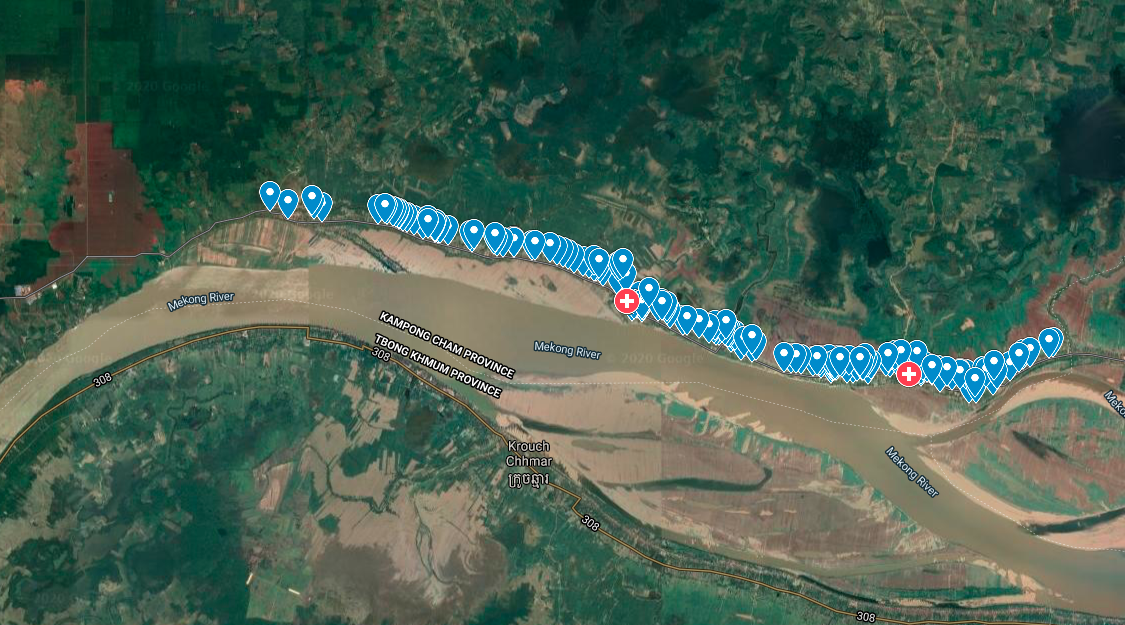
Supplemental figure. Map of study villages surrounded by farmland and the Mekong river.**

Health Centre

Household
